# Identification of novel differentiation trajectories and gene network associations with ectopic pregnancy in fallopian tube epithelium

**DOI:** 10.1093/humrep/deaf200

**Published:** 2025-11-03

**Authors:** Lily I Wright, Ivan Wangsaputra, Terence Garner, Megan C Sharps, Roger Sturmey, Peter T Ruane, Adam Stevens

**Affiliations:** Maternal and Fetal Health Research Centre, Division of Developmental Biology and Medicine, School of Medical Sciences, Faculty of Biology, Medicine, and Health, University of Manchester, Manchester, UK; Maternal and Fetal Health Research Centre, Division of Developmental Biology and Medicine, School of Medical Sciences, Faculty of Biology, Medicine, and Health, University of Manchester, Manchester, UK; Maternal and Fetal Health Research Centre, Division of Developmental Biology and Medicine, School of Medical Sciences, Faculty of Biology, Medicine, and Health, University of Manchester, Manchester, UK; Maternal and Fetal Health Research Centre, Division of Developmental Biology and Medicine, School of Medical Sciences, Faculty of Biology, Medicine, and Health, University of Manchester, Manchester, UK; Biomedical Institute for Multimorbidity, Hull York Medical School, University of Hull, Hull, UK; Maternal and Fetal Health Research Centre, Division of Developmental Biology and Medicine, School of Medical Sciences, Faculty of Biology, Medicine, and Health, University of Manchester, Manchester, UK; Maternal and Fetal Health Research Centre, Division of Developmental Biology and Medicine, School of Medical Sciences, Faculty of Biology, Medicine, and Health, University of Manchester, Manchester, UK

**Keywords:** fallopian tube, endometrium, tubal ectopic pregnancy, implantation, single-cell transcriptomics, network modelling, hypergraph

## Abstract

**STUDY QUESTION:**

Can network modelling of single-cell transcriptomic data identify cellular developmental trajectories of fallopian tube (FT) epithelium and reveal functional and pathological divergence from the endometrium?

**SUMMARY ANSWER:**

A bidirectional differentiation trajectory originating from a novel OVGP1+ progenitor population of FT epithelial cells was uncovered, and causal network modelling of whole-transcriptome activity in the FT and endometrium revealed functional divergence between their secretory epithelial cells, with implications for ectopic pregnancy candidate genes.

**WHAT IS KNOWN ALREADY:**

The FT forms the *in vivo* peri-conceptual environment, which has a significant impact on programming offspring health. The FT epithelium establishes this environment; however, the epithelial cell types are poorly characterized in health and disease.

**STUDY DESIGN, SIZE, DURATION:**

Publicly available, benign FT single-cell RNA sequencing (scRNA-seq) samples from 13 women across three previous studies were combined. Endometrial scRNA-seq samples from 13 women from one study were used to demonstrate transcriptomic differences between the epithelia of the two tissues. Network models of transcriptomic action were constructed with hypergraphs.

**PARTICIPANTS/MATERIALS, SETTING, METHODS:**

A meta-analysis of FT scRNA-seq samples was performed to identify epithelial populations. Differential gene expression assessed differences between FT and endometrial epithelial scRNA-seq data. Functional differences between secretory cells in the tissues were characterized using hypergraph models. To identify associations with ectopic pregnancy, expression quantitative trait loci (eQTLs) from a recent GWAS were mapped onto the network models.

**MAIN RESULTS AND THE ROLE OF CHANCE:**

Epithelial cells (n = 14 360) were clustered into eight secretory and ciliated epithelial populations in the meta-analysis of three scRNA-seq datasets. A novel *OVGP1*+ epithelial progenitor cell was also identified, and its bidirectional differentiation to mature secretory or mature ciliated populations was mapped by RNA velocity analysis. This progenitor exhibited a high velocity magnitude (12.47) and low confidence (0.69): a combination strongly indicative of multipotent progenitor status. Comparing FT epithelial cells with endometrial epithelial cells revealed 5.3-fold fewer shared genes between FT and endometrial glandular secretory cells than between FT and endometrial ciliated cells, suggesting functional divergence of secretory cells along the reproductive tract. Hypergraphs were used to identify highly coordinated regions of the transcriptome robustly associated with functional gene networks. In the FT secretory cells, these networks were enriched for lipid-related (false discovery rate (FDR) < 0.002) and immune-related (FDR < 0.00007) pathways. We mapped eQTLs from a GWAS meta-analysis of 7070 women with ectopic pregnancy over a range of significance (*P* = 1.68 × 10^−21^–5.8 × 10^−4^) to the hypergraphs of FT and endometrium. Of the 22 genes present in the hypergraphs, 13 of these clustered as highly coordinated genes. This demonstrated the functional importance of *MUC1* in the FT and endometrium (GWAS Study *P* = 5.32 × 10^−9^) and identified additional genes (*SLC7A2*, *CLDN1*, *GLS*, *PEX6*, *PLXNA4*, *NR2F1*, *CLGN*, *PGGHG*, and *ANKRD36*) implicated in ectopic pregnancy and eutopic pregnancy.

**LIMITATIONS, REASONS FOR CAUTION:**

The sample size of reproductive age women was limited in previous studies, and though causal network modelling was used and previous mechanistic data supports candidate gene involvement, no *in vitro* or *in vivo* validation of candidate was performed.

**WIDER IMPLICATIONS OF THE FINDINGS:**

These findings consolidate the existing single-cell transcriptomic datasets of the FT to provide a comprehensive understanding of epithelial populations and define functionally distinct secretory cells that contribute to the peri-conceptual environment of the FT. This study further implicates the role of MUC1 and secretory cells in ectopic pregnancy and suggests future targets for investigating embryo implantation in the FT and endometrium.

**STUDY FUNDING/COMPETING INTEREST(S):**

No funding was received for this study. The authors do not disclose any competing interests.

**TRIAL REGISTRATION NUMBER:**

N/A.

## Introduction

The fallopian tube (FT) is the site of early reproductive processes *in vivo*, facilitating gamete transport, fertilization, and pre-implantation embryo development. The peri-conceptual environment provided by the FT has a critical hand in programming offspring health, as underscored by metabolic deficiency in animal studies and assisted reproductive treatment outcomes in humans (Fleming *et al.*, 2018; Hann *et al.*, 2018). Moreover, FT dysfunction can cause infertility and ectopic pregnancy (Refaat *et al.*, 2012; Abrao *et al.*, 2013). Despite this fundamental role in reproductive health and developmental programming, the biological mechanisms underpinning FT function in health and disease remain underexplored (Li and Winuthayanon, 2017; Csobonyeiova *et al.*, 2022).

The FT is divided into four regions: distally, the fimbriae, infundibulum, and ampulla, and proximal to the uterus, the isthmus. It is composed of a thin, outer smooth muscle layer, the myosalpinx, and the endosalpinx, which consists of stroma and a simple columnar epithelium of secretory and multi-ciliated cells lining the lumen. The myosalpinx and ciliated epithelium combine to move tubal fluid along the tract, and the secretory cells of the epithelium produce secretions, which provide the peri-conceptual environment for gametes and preimplantation embryos (Crow *et al.*, 1994; Leese *et al.*, 2001). FT fluid has been studied *ex vivo* and *in vivo*, demonstrating an effect on embryo metabolism and DNA methylation (Leese *et al.*, 2008; Barrera *et al.*, 2013, 2017). Moreover, recent *in vivo* work in mice confirms the role of ciliated cells in moving fluid, thus contributing to gamete/embryo transport (Wang and Larina, 2021, 2023).

To highlight the FT as a distinct functional tissue, we compare FT and endometrial epithelial cells in the secretory phase. The FT and uterus share common embryological origins and exist as a continuum; however, despite their anatomical proximity, they have significantly different functions in reproduction (Sajjad, 2010; Mullen and Behringer, 2014). Unlike the endometrium, the FT lumen does not undergo a receptivity period, yet it is susceptible to embryo implantation in 1–2% of pregnancies (Barnhart, 2009). Tubal ectopic pregnancy is a common but serious condition, though subsequent placentation is not viable; it can cause severe haemorrhage when undetected.

Several single-cell RNA sequencing (scRNA-seq) datasets investigating ovarian cancer and hydrosalpinx have characterized cell types of the FT epithelium (Hu *et al.*, 2020; Dinh *et al.*, 2021; Ulrich *et al.*, 2022). These datasets highlight alternate hypotheses of epithelial differentiation in the FT. Dinh *et al.* (2021) modelled two differentiation pathways, where secretory progenitor cells give rise to a mature secretory population and where an alternate ‘epithelial to mesenchymal transformation’ cell type gives rise to both ciliated epithelial cells and stromal cells. Conversely, Ulrich *et al.* (2022) described secretory cells differentiating to ciliated cells via a highly proliferative transitioning population.

Single-cell transcriptome datasets also enable the analysis of developmental trajectories and causal modelling, allowing us to infer functional and pathological gene networks within cell populations of the FT. By constructing hypergraphs from gene expression matrices, we can build gene network models, which capture relationships among groups of genes rather than pairs of genes (Johnson, 2011). Identifying these higher-order gene interactions allows us to identify genes with a high number of connections to the rest of the transcriptome, suggesting their functional biological significance (Battiston *et al.*, 2021). Hypergraph models have been shown to capture biological relevance and causality in omics datasets of embryo implantation (Ruane *et al.*, 2022), rare disease (Cuvertino *et al.*, 2024), and developmental programming (Ripley *et al.*, 2023).

Here, we perform a meta-analysis of healthy FT scRNA-seq data from 13 women to examine FT epithelial cell sub-populations and differentiation in greater depth and identify clinically relevant functional differences between FT and endometrium in the context of tubal ectopic pregnancy.

## Materials and methods

### scRNA-seq data and samples

Published FT scRNA-seq FASTQs were obtained from GSE183837 (Hu *et al.*, 2020), GSE151214 (Dinh *et al.*, 2021), and GSE178101 (Ulrich *et al.*, 2022). Processed FT validation dataset was obtained from CZ CELLxGENE (Weigert *et al.*, 2025). Published endometrial scRNA-seq FASTQs were obtained from E-MTAB-10287 (Garcia-Alonso *et al.*, 2021). Cadaveric samples and patients with diagnosed malignancies were excluded. FT data from 13 patient samples and endometrial data from 13 patient samples were included in the final data analysis. Publicly available demographics are shown in Supplementary Table S1.

### Raw data processing

Raw data processing was performed using a standardized methodology. FASTQ files were processed using Cell Ranger (7.1.0) analysis pipeline and aligned to GRCh38 reference genome to generate single-cell gene expression count files (51). For GSE183837 (Hu *et al.*, 2020), FASTQ files were processed using HISAT2 (2.2.1) and SAMtools (1.9). Velocyto (0.17) was used to generate all unspliced and spliced count matrices for RNA velocity analysis (La Manno *et al.*, 2018).

### scRNA-seq analysis

scRNA-seq data analyses were performed using single-cell gene expression count files in Python (3.11.4). Scanpy package (1.9.3) was used for all preprocessing, quality control, dimensionality reduction, visualization, and marker gene identification steps (Wolf *et al.* 2018).

### Filtering, preprocessing, and dimensionality reduction

For all data analysis, cells with <200 genes expressed, and genes expressed in <3 cells were removed, following standard parameters of the Scanpy package (Wolf *et al.*, 2018). Cells with >850 000 total gene counts, >30% mitochondrial gene counts, and >8000 genes in the count matrix were also removed. To batch correct and integrate multiple datasets, datasets were concatenated, highly variable genes were selected with a batch factor of patient, and ComBat (Zhang *et al.*, 2020) batch correction was used with the dataset as a batch factor.

For individual FT datasets, dimensionality reduction and clustering, nearest neighbour graphs were constructed using 8 principal components and 10 nearest neighbours. Leiden clustering was performed between 0.4 and 0.5 resolution. To produce the combined FT cellular atlas, raw count files of individual FT datasets were concatenated and then filtered and pre-processed. The nearest neighbours’ graph was constructed using 9 principal components and 10 neighbours; Leiden clustering was run at 1.7 resolution. FT epithelium was sub-clustered by cropping data to only ciliated and secretory epithelial cells. Dimensionality reduction and clustering were re-computed, and the nearest neighbours’ graph was constructed using 7 principal components and 90 neighbours. For the FT validation dataset used, epithelial cell types were subset, and nearest neighbour graphs were constructed using 6 principal components and 10 neighbours.

To resolve endometrial cell clusters, a nearest neighbours’ graph was constructed with 15 principal components and 300 neighbours. Epithelium was sub-clustered by cropping data to only Sox9+ Epithelial, Luminal Epithelial, Glandular Epithelial, and Ciliated cells. Dimensionality reduction and clustering were re-computed, and a nearest neighbours’ graph was constructed with 25 principal components and 200 neighbours. Leiden clustering was run at Resolution 1.

To compare endometrial and FT epithelial cells, epithelial datasets were concatenated and batch corrected, and dimensionality reduction was re-computed. The nearest neighbours’ graph was constructed with 25 principal components and 200 neighbours. The endometrial and FT full cellular atlas was constructed by concatenating raw datasets with batch correction; dimensionality reduction and clustering were computed to identify cell types. A nearest neighbours’ graph was constructed with 25 principal components and 250 neighbours. Leiden clustering was run at Resolution 2.

In the FT, major cell types and epithelial sub-populations were annotated by inspection of literature-based marker genes and cluster-defined marker genes. In the endometrium, cell types were annotated using the marker genes reported by Garcia-Alonso *et al.* (2021). Differentially expressed marker genes were identified for each cluster using Wilcoxon test. Proportional Venn diagrams were generated using BioVenn (Hulsen *et al.*, 2008).

### Trajectory inference

ScVelo package (0.2.5) was used for RNA velocity analysis plotting functions and computing velocity magnitude and confidence (Bergen *et al.*, 2020). Monocle3 (1.4.23) was used for the FT validation dataset (Weigert *et al.*, 2025) where spliced and un-spliced mRNA counts could not be obtained. Scanpy’s diffusion pseudotime was used to compare gene expression across ciliated and secretory differentiation trajectories in the FT dataset and the validation FT dataset.

### Statistical analysis

Linear regression was used to compare proportions of epithelial sub-populations between menstrual cycle phases in FT and endometrial epithelium in GraphPad Prism (9.5.1). Differential gene expression was tested using the Wilcoxon rank sum in Scanpy (1.9.3).

### Hypergraph analysis

**Figure 1.**
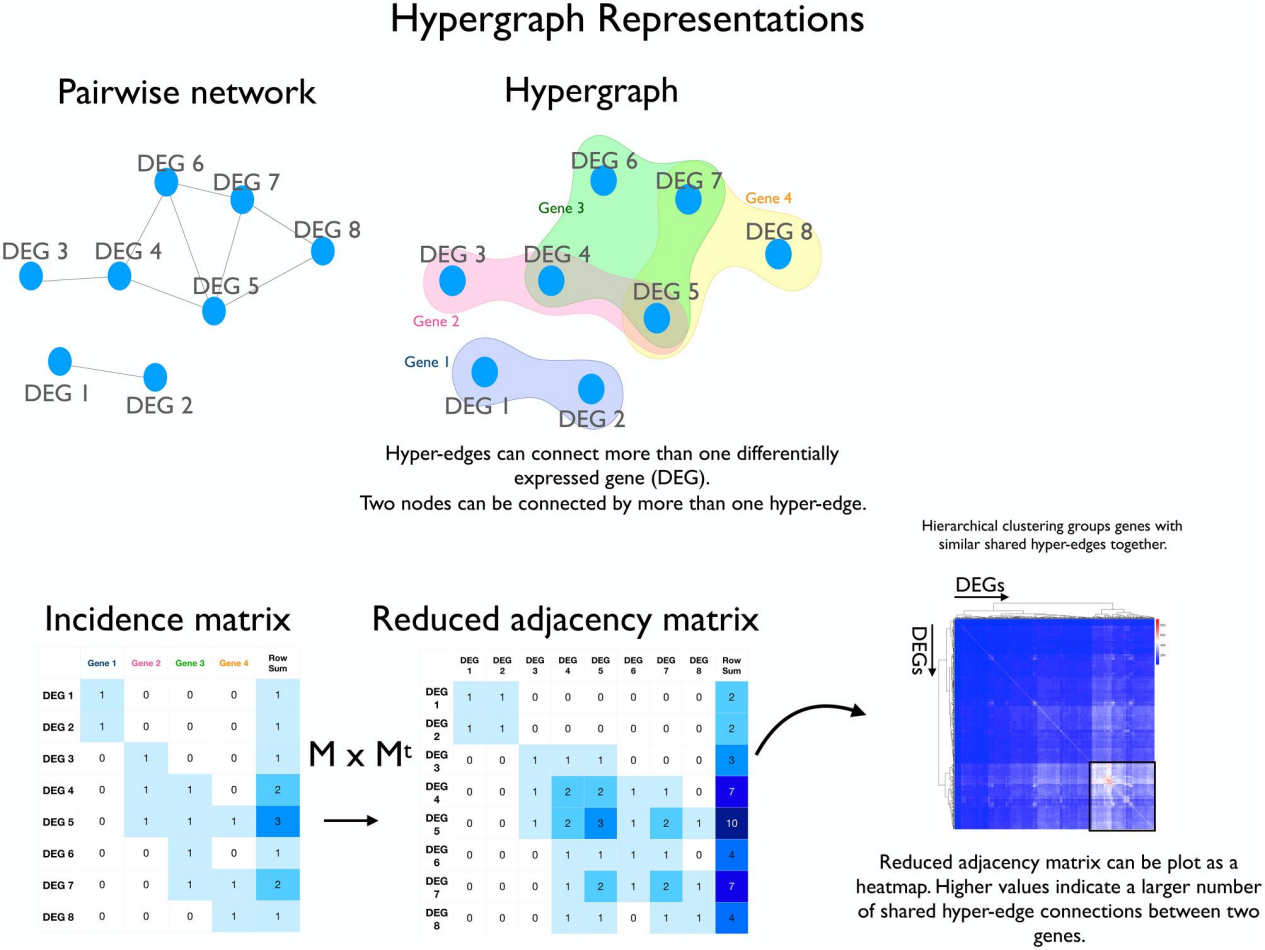
**Hypergraph representations**. A traditional pairwise network is shown alongside a hypergraph, where two nodes can be connected by more than one edge. Hypergraphs can be represented as an incidence matrix or by a reduced adjacency matrix, which can also be plotted as a heatmap.

Hypergraphs were generated to investigate functional relationships in the differentially expressed genes between the FT and endometrial epithelia, and between the rest of the transcriptome in the FT, glandular, and luminal endometrium. All hypergraph analyses were performed in R. Correlation matrices were constructed using the top 600 differentially expressed genes (DEGs) between secretory-phase secretory epithelial cells of the FT and endometrium as nodes, and the remaining genes from the integrated full cellular atlas of either the FT, glandular or luminal endometrium as edges. Then 600 genes were selected as a meaningful number of DEGs by identifying the inflection point in the ranked −log_10_ (*P*-value) distribution of DEGs. Hypergraphs are represented as both incidence matrices and reduced adjacency matrices (Fig. 1).

The standard deviation of the correlation matrices was calculated to set a threshold against which correlation matrices were binarized. Matrix multiplication was performed to generate an adjacency matrix where values represent the number of correlations shared between the differentially expressed genes and genes from the rest of the transcriptome for each tissue. These were plotted as heatmaps where higher counts indicated a higher number of correlations. Hypergraph construction is detailed in Supplementary Fig. S1. Hierarchical clustering of the DEGs was performed to identify central clusters of genes with the highest number of correlations, suggesting groups of functionally active genes. Central clusters of functionally active genes were identified for each hypergraph constructed from FT, luminal, or glandular epithelium. To compare functionally active genes between the three tissue epithelia, common and unique genes of the respective central clusters were identified, and proportional Venn diagrams were constructed. Random networks were generated by randomizing clique reduction graphs over 1000 iterations.

### Gene ontology

Webgestalt (Liao *et al.*, 2019) over-representation analysis of the GeneOntology ‘Biological Process’ database was used to assess hypergraph central clusters for each tissue. Default parameters were used. False discovery rate and *P* values were presented as −log10 using R.

### Tubal ectopic pregnancy genome-wide association

To identify genes associated with ectopic pregnancy that retain relevance but did not reach the *P*-value defined by the GWAS study (Chen *et al.*, 2021), GCST90272883 (Pujol Gualdo *et al.*, 2023) GWAS summary statistics were downloaded from the GWAS catalogue. To identify genes where expression is altered, variantIDs of the lowest 10 000 *P*-values were called from GTEx Portal API V2 ‘singleTissueEqtlByLocation’, accessed using Python (3.11.4). All human tissues bar the nervous system were searched. Summary statistic *P*-values of the GWAS genes identified in the 600 differentially expressed hypergraph genes were compiled in a violin plot.

To determine the significance of ectopic-associated GWAS genes across the three tissue hypergraphs, row sums, or degree centrality, of each DEG were calculated. This represents the number of direct connections with the rest of the transcriptome. Row sums were ranked, using an average for split ranks. Ranks of GWAS genes were identified and plotted for each tissue.

Additional disease traits were identified using the phenome-wide association gene ATLAS of 452 264 UK Biobank White British individuals (Canela-Xandri *et al.*, 2018). Reference SNP cluster IDs (rsIDs) of *MUC1*, *SLC7A2*, *HLA-DRB5*, and *HLA-DRB1* SNPs present in the ectopic pregnancy GWAS were searched to identify associations per variant. All significant associations (*P* < 0.05) relating to pregnancy and reproductive outcomes were recorded.

### Data and code availability

FT scRNA-seq datasets are available at GEO under GSE183837 (Hu *et al.*, 2020), GSE151214 (Dinh *et al.*, 2021), and GSE178101 (Ulrich *et al.*, 2022). The endometrial scRNA-seq dataset is available at ArrayExpress under E-MTAB-10287 (Garcia-Alonso *et al.*, 2021). Code is available at: https://github.com/adam-stevens-lab/fallopiantube

## Results

### Meta-analysis of FT single-cell RNA-seq datasets

**Figure 2.**
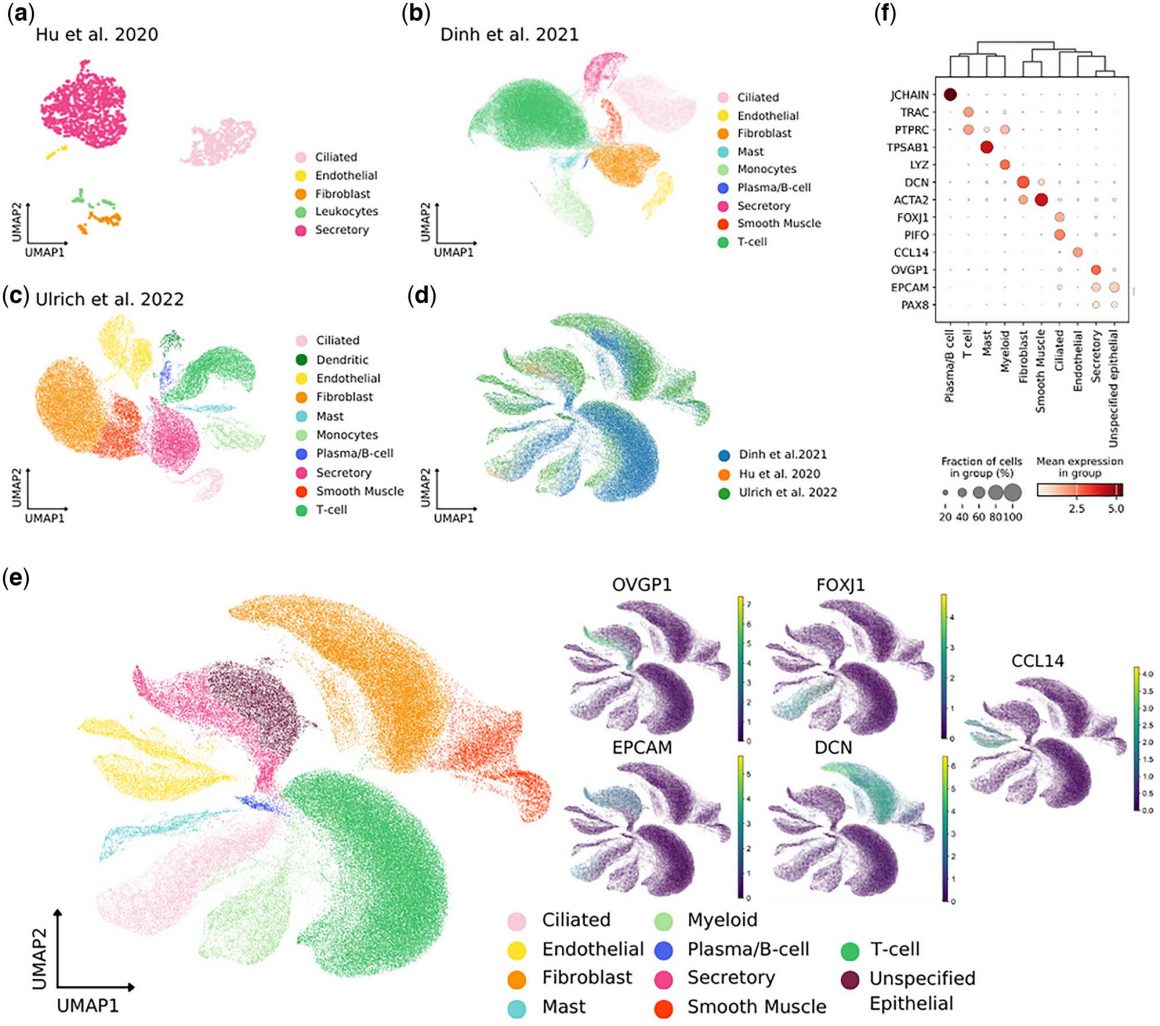
**Meta-analysis of fallopian tube (FT) scRNA-seq datasets**. (**a**) UMAP projection of cell types in Hu *et al.* scRNA-seq dataset. (**b**) UMAP projection of cell types in Dinh *et al.* scRNA-seq dataset. (**c**) UMAP projection of cell types in Ulrich *et al.* scRNA-seq dataset. (**d**) UMAP projection of three integrated FT scRNA-seq datasets, coloured by Author. (**e**) UMAP projection of three integrated FT scRNA-seq datasets, coloured by cell type; cell-type marker gene expression is shown: OVGP1, secretory epithelial cell; FOXJ1, ciliated epithelial cell; CCL14, endothelial cell; DCN, fibroblast; EPCAM, epithelial cell. (**f**) Dotplot illustrating mean gene expression and fraction of cells expressing marker genes in key cell types of the integrated FT meta-analysis. UMAP, Uniform Manifold Approximation Projection; FT, fallopian tube; scRNA-seq, single-cell RNA sequencing.

Initial clustering of individual healthy FT datasets of pre- and post-menopausal samples was performed to assess concordance of cell types, identifying 5 distinct cell populations in the Hu *et al.* (2020) dataset, 10 in the Dinh *et al.* (2021) dataset, and 9 in the Ulrich *et al.* (2022) dataset (Fig. 2a–c). The Dinh and Ulrich datasets contained the expected immune populations, secretory and ciliated epithelia, and supporting fibroblasts, endothelial, and smooth muscle cells. The samples used in the Hu dataset were sorted by flow cytometry to select epithelial cells for sequencing, meaning that minimal immune and supporting cells are present.

To perform a meta-analysis of FT scRNA-seq data, the three raw datasets were integrated to a combined matrix of 142 339 cells (Fig. 2d and e). Clustering identified nine major cell types, which were annotated according to marker gene expression: four immune cell populations, Plasma/B cells (*JCHAIN*+), T-cells (*TRAC*+), Mast cells (*TPSAB1*+), Myeloid cells (*LYZ*+), as well as Fibroblasts (*DCN*+), Endothelial (*CCL14+*) cells, Smooth Muscle cells (*ACTA2*+), and Secretory (*OVGP1+*) and Ciliated (*FOXJ1+*) epithelial cells (Fig. 2f). A large population of cells were marked by classical epithelial markers (E*PCAM*, *PAX8*, *KRT18*, *MMP7*) but were *OVGP1*-negative and so were labelled as Unspecified Epithelial. To understand FT regulation of the peri-conceptual environment, we set out to characterize epithelial cells in more depth.

### The FT epithelium contains eight populations of ciliated, secretory, and progenitor cells

**Figure 3.**
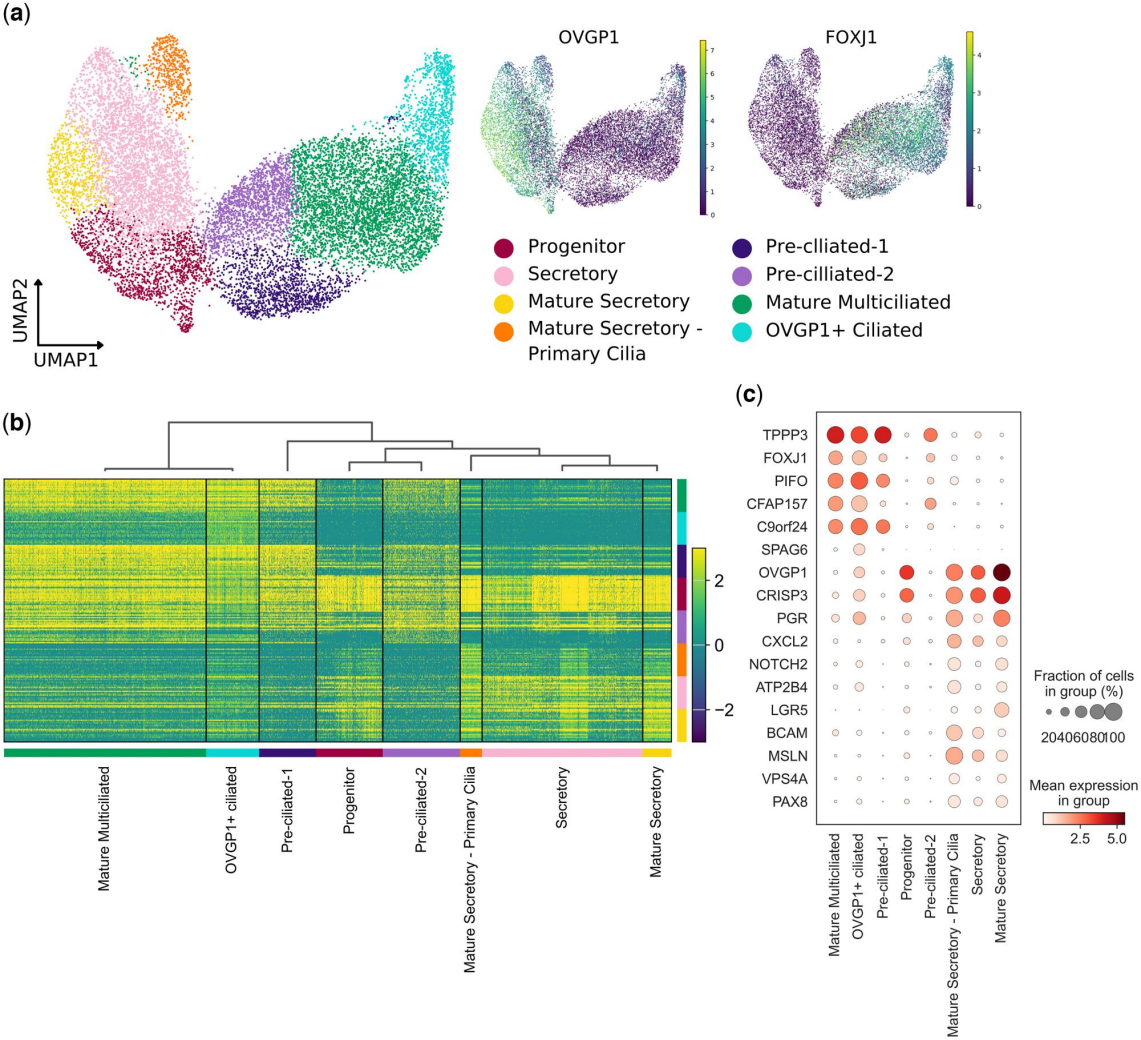
**Clusters of 14 630 fallopian tube (FT) epithelial cells in three secretory, one progenitor, and four ciliated populations**. (**a**) UMAP projection of FT epithelial cells from Hu *et al.*, Ulrich *et al.*, Dinh *et al.*, datasets, showing eight populations, and UMAP of OVGP1 expression marking secretory cells and FOXJ1 expression marking ciliated cells. (**b**) Heatmap of the top 25 marker genes for each cell population, with genes represented on the *x*-axis and clusters of cell populations on the *y*-axis. (**c**) Dotplot illustrating mean gene expression and fraction of cells expressing key marker genes in populations of FT epithelial cells. UMAP, Uniform Manifold Approximation Projection; FT, fallopian tube.

To focus on epithelial cells, these cells were subset and re-clustered. Eight cell types were identified among the 14 360 epithelial cells (Fig. 3a and b): (i) Mature Secretory, marked by high expression of *OVGP1* and a secretory marker, *LGR5* (LGR*5+*, *OVGP1+*) (Fig. 3c); (ii) Secretory, which lacked LGR5 expression (*OVGP1+*, *LGR5*−); (iii) Mature Secretory—Primary cilia, which expressed *OVGP1* and *PIFO*, a marker of primary cilia, but did not express *FOXJ1*, a multi-ciliation marker (*OVGP1+*, *PIFO+*, *FOXJ1−*, *SPAG6+*); (iv) Mature Multiciliated, marked by classical ciliation marker FOXJ1 and loss of OVGP1 expression (*FOXJ1+*, *OVGP1−*, *PIFO+*); (v) OVGP1+ Ciliated *(FOXJ1+*, *OVGP1+*, *PIFO+*); (vi) Pre-ciliated-2 (*PIFO+*, *CFAP157−*, *C9orf24+*, *OVGP1−*); (vii) Pre-ciliated-1 (*PIFO+*, *CFAP157+*, *C9orf24−*, *OVGP1−*), both marked by lower levels of *FOXJ1* than mature multiciliated clusters, and an absence of ciliary genes, such as *CFAP157* or *C9ORF24*, suggesting they may be developing ciliated cells; and (viii) Progenitor, marked by OVGP1 expression and the absence of Secretory markers such as PAX8 (*OVGP1+*, *PAX8−*, *FOXJ1−*, *PIFO−*). Full marker genes are shown in Supplementary Table S2. This allowed us to conclude that epithelial cells in the human FT can be assigned into one of three Mature Secretory populations, or four Ciliated populations, or a Progenitor population.

### RNA velocity analysis suggests a bidirectional development of mature secretory and ciliated epithelial cells in the FT epithelium

**Figure 4.**
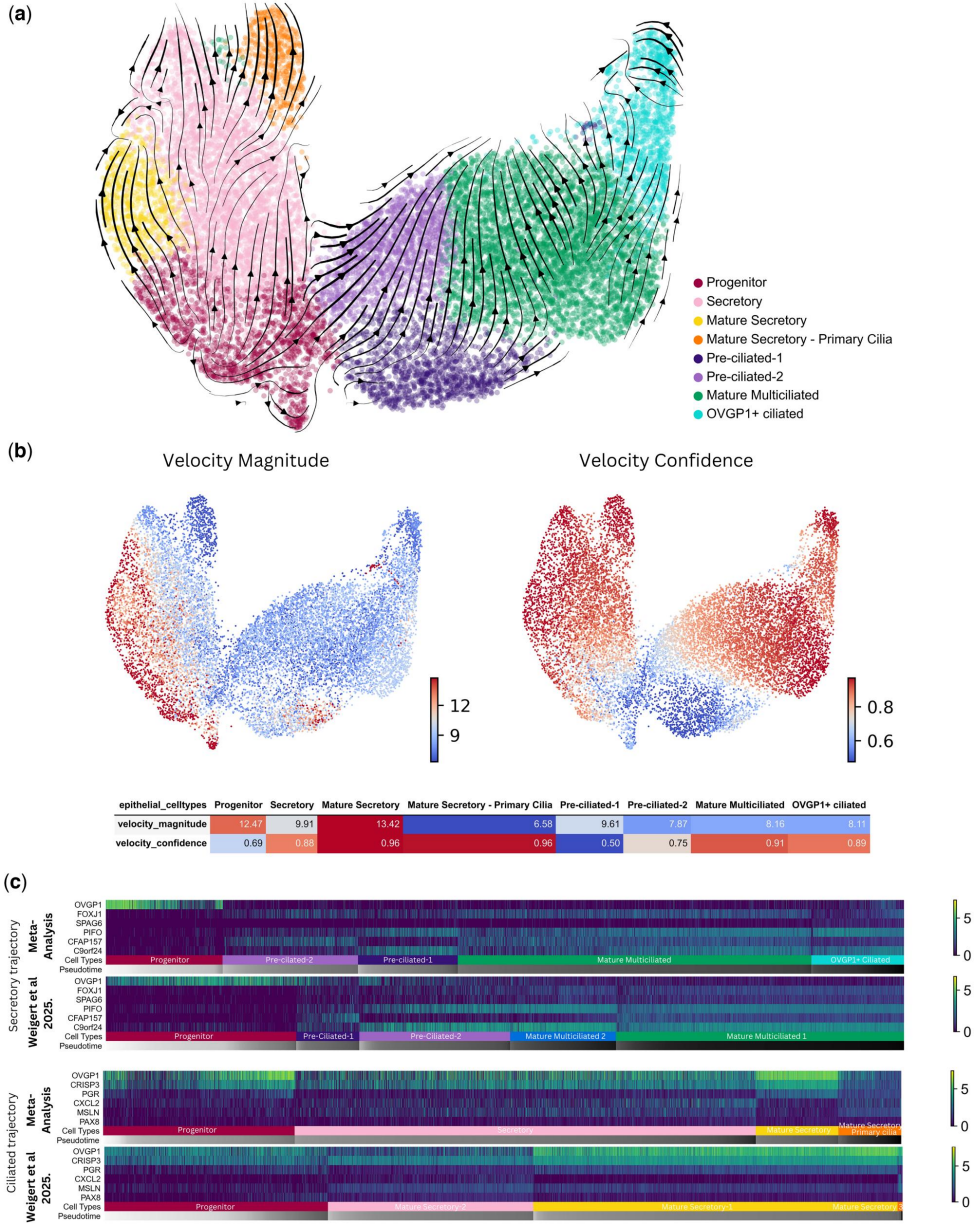
**RNA velocity analysis of fallopian tube (FT) epithelial cells**. (**a**) Estimated RNA Velocity graph projected onto UMAP of FT epithelial cell populations; velocity vectors are visualized as streamlines. (**b**) UMAP projections coloured by Velocity Magnitude, a measure of differentiation speed, and Velocity Confidence, a measure of agreement between the velocity vector of an individual cell and its neighbouring cell; for each cell, both values were calculated per cell using scVelo. The table shows the average velocity length and velocity confidence for each FT epithelial cell population. (**c**) Gene expression across diffusion pseudotime for ciliated and secretory differentiation trajectories in the meta-analysis and validation datasets. UMAP, Uniform Manifold Approximation Projection; FT, fallopian tube.

Next, the differentiation trajectory of FT epithelia was assessed. Using RNA velocity analysis (Bergen *et al.*, 2020), a velocity vector is calculated for each cell, and the future state of the cell can be inferred from the direction of the velocity vector. By plotting these velocity vectors, it was possible to map a bidirectional developmental trajectory with the progenitor population acting as a source, differentiating into terminal Mature Secretory or Ciliated populations (Fig. 4a).

Velocity magnitude, a measure of differentiation speed, and velocity confidence, a measure of coherence between the velocity vectors of an individual cell and its neighbouring cells, were also computed (Fig. 4b). Higher velocity confidence is seen in Mature Ciliated and Mature Secretory populations, suggesting a cohesive differentiation trajectory, as expected for differentiated cell types (Furusawa and Kaneko, 2009; Rue and Martinez Arias, 2015). Developing Pre-Ciliated populations have a lower velocity confidence and a lower velocity magnitude, whilst the Progenitor population had both the lowest velocity confidence and the highest velocity magnitude for one population, suggesting chaotic and less coherent gene expression dynamics, consistent with that observed in other progenitor cell niches *in vivo* (Furusawa and Kaneko, 2009; Rue and Martinez Arias, 2015).

To validate this novel progenitor population and differentiation trajectory, we analysed pre-menopausal epithelial cells from a fourth healthy pre-menopausal FT dataset (Weigert *et al.*, 2025). The cell types defined in our epithelial meta-analysis were recapitulated in Uniform Manifold Approximation Projection (UMAPs) generated of the Weigert dataset (Supplementary Fig. S2a and b) and showed highly similar marker gene expression (Supplementary Fig. S2c). Owing to the absence of splicing count data, the bidirectional differentiation trajectory was constructed using Monocle3 (Supplementary Fig. S2d), with the progenitor population giving rise to both Secretory and Ciliated cell types. A third cell trajectory metric, diffusion pseudotime was used to compare marker gene expression across both ciliated and secretory developmental trajectories in the epithelial meta-analysis and the Weigert validation dataset (Fig. 4c). Highly similar transitions in gene expression were observed between the two datasets, validating our novel bidirectional trajectory.

### A comparison of FT and endometrial epithelial cells reveals transcriptome differences between the tissues

**Figure 5.**
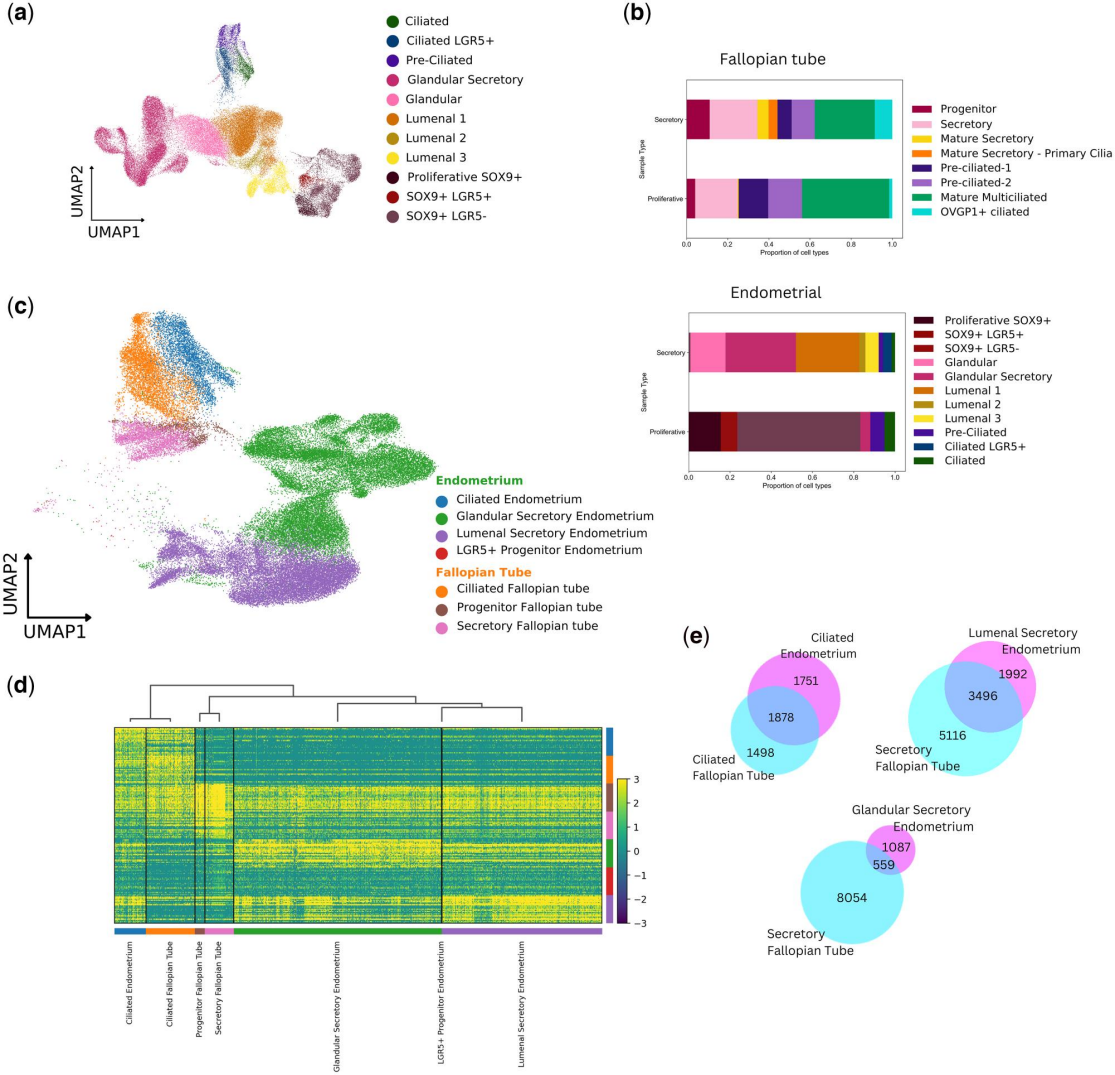
**A comparison of 57 951 secretory phase fallopian tube (FT) and endometrial epithelial cells**. (**a**) UMAP of epithelial cell populations isolated from Garcia-Alonso *et al.* Endometrial scRNA-seq dataset using scanpy workflow. (**b**) Proportions of epithelial cell populations in the secretory and proliferative phases in the endometrial scRNA-seq dataset and the FT scRNA-seq dataset. (**c**) UMAP projection of integrated endometrial and FT secretory phase epithelium, coloured by epithelial populations. (**d**) Heatmap of the top 25 differentially expressed genes (DEGs) for each epithelial population, with genes presented on the *x*-axis and clusters of epithelial cell types on the *y*-axis. (**e**) Venn diagrams of significant DEGs (*P* < 0.05) in ciliated and secretory epithelial cells of FT and endometrium. UMAP, Uniform Manifold Approximation Projection; FT, fallopian tube; DEG, differentially expressed gene.

We next sought to identify the features that demarcate the FT from the endometrium, the developmental sister tissue, and nearest anatomical neighbour. A total of 58 463 endometrial epithelial cells were isolated from an scRNA-seq dataset of healthy, pre-menopausal endometrial samples (Garcia-Alonso *et al.*, 2021). Epithelial populations were annotated based on the Garcia-Alonso *et al.* (2021) original data analysis (Fig. 5a).

Differences in the epithelial populations between secretory and proliferative phases were assessed between the FT and endometrium (Fig. 5b). Proportions of epithelial populations were similar between the proliferative and secretory phases in the FT (R2 = 0.800, *P* = 0.003), unlike in the endometrium, where epithelial cell proportions change dramatically across the menstrual phase (R2 = 0.096, *P* = 0.354) (Supplementary Fig. S3). In the FT epithelium, Mature Secretory cells and *OVGP1+* Ciliated cells emerge in the secretory phase, suggesting a hormonal influence on differentiation.

Given these differences between menstrual phases, we integrated secretory epithelial cells from the two tissues to develop a UMAP of 57 951 cells from 16 individuals. Clustering the integrated FT and endometrial dataset revealed that epithelial populations were well preserved (Supplementary Fig. S4). To compare epithelial cells between the tissues, populations were grouped into seven key categories: (i) Ciliated Endometrium; (ii) Glandular Secretory Endometrium; (iii) Luminal Secretory Endometrium; (iv) LGR5+ Progenitor Endometrium; (v) Ciliated FT; (vi) Progenitor FT; and (vii) Secretory FT. FT and endometrial ciliated epithelial cells clustered together in UMAP projections and dendrogram clustering, suggesting similarity in global gene expression. However, secretory epithelial cells remained distinct between the two tissues (Fig. 5c and d), suggesting transcriptomic differences between the secretory epithelial types of the endometrium and the FT.

Comparing significant differential gene expression (*P* ≤ 0.05) between epithelial cell categories revealed that 8054 and 5116 genes are significantly differentially expressed in the FT secretory cells compared to endometrial glandular secretory cells and luminal secretory cells, respectively. In contrast, only 1498 genes were significantly differentially expressed in ciliated FT cells compared to ciliated endometrial cells (Fig. 5f) (Supplementary Table S3).

### Functional similarities and differences between epithelial secretory cells in FT and endometrium

This divergent gene expression between FT and endometrial secretory cells is suggestive of functional differences. Similarities in gene expression of ciliated cells between tissues also suggest that key differences between mucosal tissues arise from secretory cells. We therefore sought to identify genes causally linked to biological function in secretory cells using higher-order network structures to model the transcriptome. Hypergraphs were constructed from secretory menstrual phase expression correlation matrices. The top 600 differentially expressed genes between secretory phase FT and endometrial secretory cells were used as node genes, and the rest of the transcriptome genes were used as edges in secretory epithelial cells of FT, glandular endometrium, and luminal endometrium (Fig. 1).

**Figure 6.**
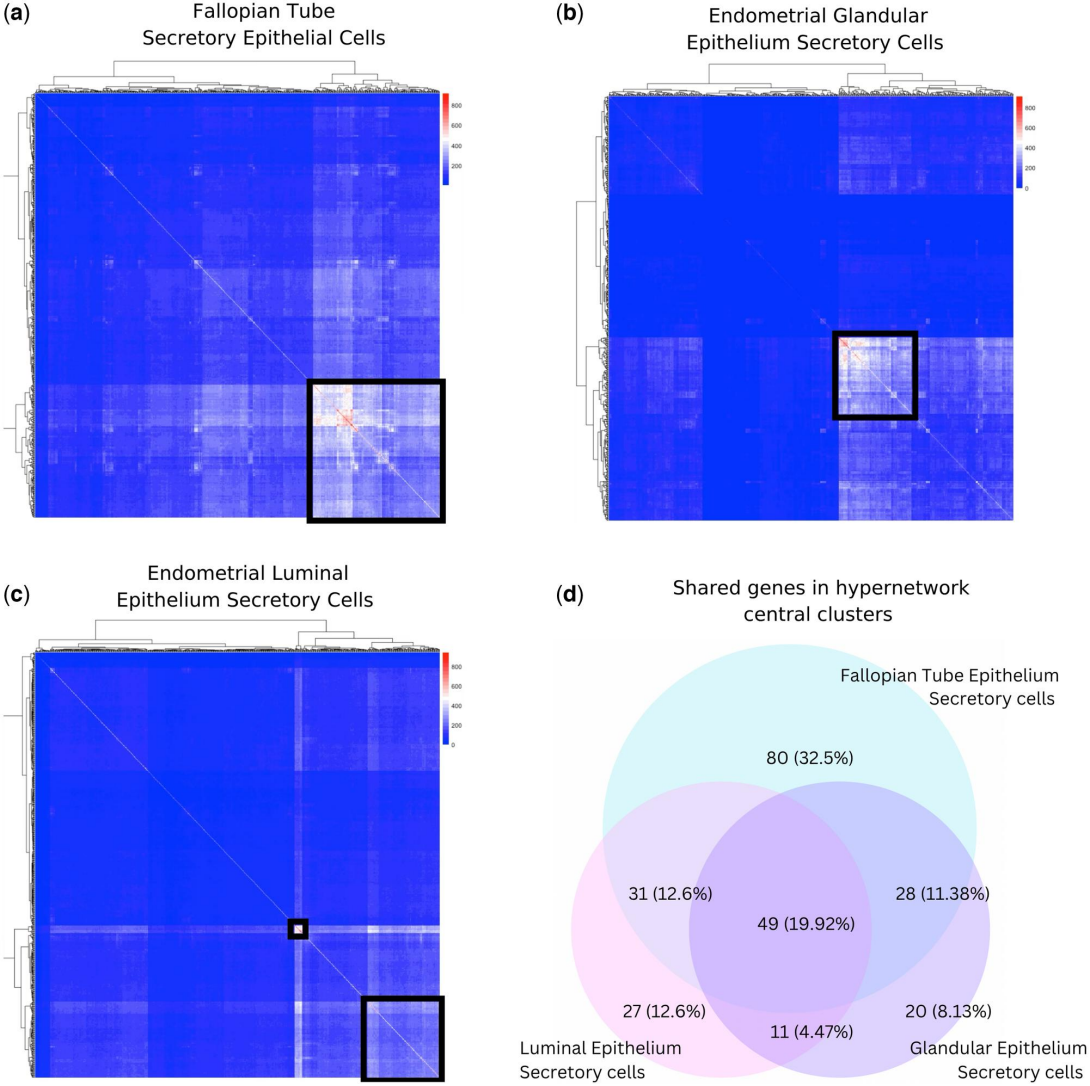
**Hypergraph analysis identifies genes with functional differences between secretory cells in fallopian tube (FT) and endometrial epithelia**. Hypergraphs constructed using the top 600 differentially expressed genes between FT and endometrial epithelial secretory cells as nodes, and the rest of transcriptome genes as nodes for: (**a**) Secretory Epithelial cells in FT, (**b**) Glandular Endometrium, and (**c**) Luminal Endometrium. Reduced adjacency matrices of hypergraphs are presented as heatmaps, where the *x* and *y* axes are DEGs; higher values indicate a greater number of shared hyperedge connections between two DEGs. (**d**) Venn diagrams indicating number and percentage of unique and shared genes between hypergraph central clusters in each tissue. FT, fallopian tube; DEG, differentially expressed gene.

Hierarchical clustering identified groups of 188 highly connected genes in the FT hypergraph, 108 genes in the glandular hypergraph, and 118 genes in the luminal epithelium hypergraph (Fig. 6a–c). Initial gene ontology over-representation analysis of these clusters suggested functional DEG networks associated with lipid metabolism and immune response biological processes distinguish FT and endometrial secretory cells (Supplementary Fig. S5). Comparisons between the tissue-specific hypergraphs revealed almost half (48.4%) of the clustered genes were shared (Fig. 6d), and the FT-specific gene set was larger (32.5%) compared to the luminal and glandular endometrium-specific subsets (12.6% and 8.1%, respectively) (Supplementary Table S4). The FT-specific gene set contained several glycoprotein genes: *OVGP1*, *LCN2*, *SRGN*, *LRG1*; genes encoding cholesterol and lipid transport-associated proteins: *APOA1*, *STARD4*, *STX11*, *SYNRG*, *TTC39B*; and several glycoprotein human leukocyte antigen class II genes: *HLA-DRB1*, *HLA-DRB5*, *HLA-DPA1*, and *HLA-DRA* (Supplementary Table S4).

### Hypergraphs identify tubal ectopic pregnancy-associated GWAS genes

To establish whether functional networks predicted in the hypergraphs could be clinically relevant to FT pathology, we evaluated tubal ectopic pregnancy-associated genes from a recent GWAS (Pujol Gualdo *et al.*, 2023). From the lowest 10 000 *P*-value SNPs of the tubal ectopic pregnancy GWAS, 1338 rsIDs mapped to expression quantitative trait loci in 22 of the 600 DEGs used to define our hypergraphs (Supplementary Fig. S6). Notably, 13 of these 22 genes clustered as the highest connected genes in the hypergraphs. To quantify the importance of these 13 tubal ectopic pregnancy GWAS genes, hypergraph row sum was calculated for each gene.

We assessed the row sums of GWAS genes in two representations of the hypergraphs: incidence matrix and clique reduction graph. The row sums of incidence matrices represent the number of transcriptome genes (edges) a DEG is incident with, whereas row sums of the clique reduction graph represent the connectivity between DEGs via shared edges (Fig. 1). As an edge can include multiple DEGs, the row sum of the clique reduction graph captures a metric of connectivity of each DEG across the higher-order structure of the hypergraph.

**Figure 7.**
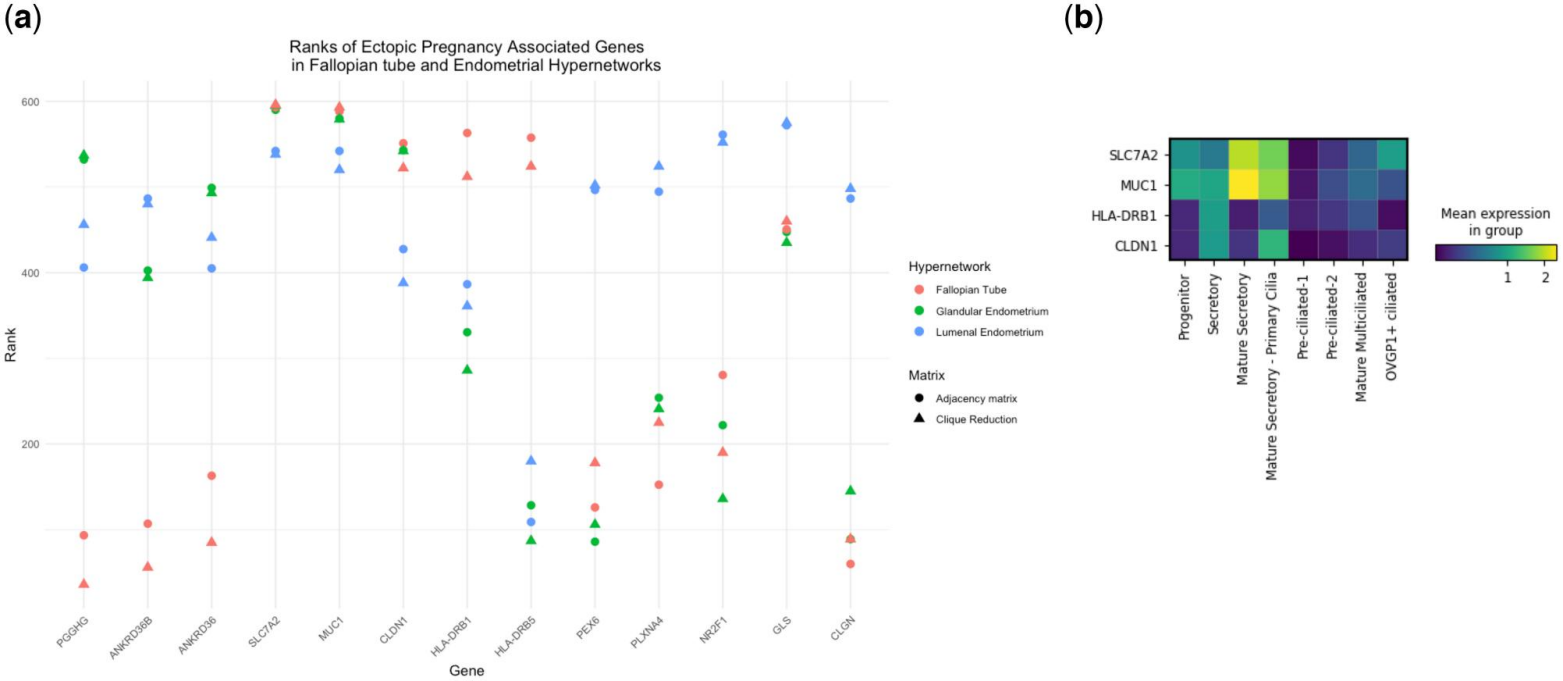
**Tubal ectopic pregnancy associated GWAS genes are ranked differentially in hypergraphs of secretory epithelial cells of the fallopian tube (FT) and glandular and luminal endometrium**. (**a**) Ranked rowsums of tubal ectopic pregnancy genes in secretory epithelial cell hypergraphs for FT, glandular endometrium and luminal endometrium, in both clique reduction and adjacency matrix hypergraph representations. (**b**) Mean Expression of the four highest ranked GWAS genes in FT epithelial populations. FT, fallopian tube; GWAS, genome-wide association study.

Several ectopic pregnancy-related GWAS genes were highly connected to the rest of the transcriptomes of all three tissues: *SLC7A2*, *MUC1*, *CLDN1*, and *GLS* (Fig. 7a). MUC1 was the strongest genetic association previously identified, thus validating high connectivity in the hypergraph as a clinically relevant functional metric. These genes are indicative of shared functionality between the tissues. *HLA-DRB1* and *HLA-DRB5* are ectopic pregnancy GWAS genes that were highly connected only in the FT secretory cell transcriptome. Additionally, *ANKRD36B*, *CLGN*, *PEX6*, *PLXNA4*, and *NR2F1* were highly connected only in the luminal endometrial secretory cells, while *ANKRD36* and *PGGHG* were highly connected in the glandular endometrial secretory cells. Row sums of these ectopic pregnancy GWAS genes were comparable between the two hypergraph representations, demonstrating a high global network connectivity (Supplementary Table S5). *MUC1* and *SLC7A2* also had the highest expression in Mature Secretory-2 FT cells (Fig. 7b). Intracellular and apical surface MUC1 protein expression has been demonstrated in the healthy fallopian epithelium in the literature (Al-Azemi *et al.*, 2009; Silva *et al.*, 2014; Brito *et al.*, 2016).

The robustness of hypergraph models of the transcriptome was assessed in each tissue by generating random network models of the transcriptome, iterated 1000 times. Row sums of GWAS genes from the transcriptome-reduced adjacency matrix were compared to those from iterated matrices and Z scores were calculated in each hypergraph. In the FT, *MUC1 Z* = 47.6; in the Glandular Endometrium, *MUC1 Z* = 66.23; and in Luminal Endometrium, *MUC1 Z* = 19.51, demonstrating significant differences in GWAS gene location in the random networks and the transcriptome hypergraphs (Supplementary Fig. S7a–c).

SNPs from the most highly connected ectopic pregnancy GWAS genes, *MUC1*, *SLC7A2*, *HLA-DRB5*, and *HLA-DRB1* were also significantly associated (*P* < 0.05) with increased and decreased risks of additional reproductive phenotypes such as ‘Pregnancy with abortive outcome’, ‘Spontaneous abortion’, and ‘Excessive, frequent and irregular menstruation’ (Supplementary Table S6).

## Discussion

The epithelial lining of the FT regulates key functions of the organ: gamete and embryo transport and pre-implantation embryo development support. Our meta-analysis of published FT scRNA-seq data characterized eight FT epithelial populations and mapped a previously unseen bidirectional differentiation of ciliated cells and specialized mature secretory cells from an *OVGP1+* progenitor population. This differentiation to mature cell types coincides with the secretory phase of the menstrual cycle. Differences between FT epithelium and that in the neighbouring endometrium were predominantly in the secretory cell populations, with hypergraph analysis identifying lipid metabolism and immune interaction pathways as functional divergences in FT secretory cells. The functional importance of hypergraph genes was underscored by their prominence in tubal ectopic pregnancy GWAS, reasserting the causal involvement of MUC1 in implantation and suggesting new genes that contribute to normal and pathological implantation.

By performing a meta-analysis of healthy FT epithelial cells, we identified eight secretory, ciliated, and progenitor cell types and mapped their bidirectional differentiation trajectory. Here, the progenitor population is characterized by *OVGP1* and *CRISP3* expression and absence of both mature secretory markers (*CXCL2*, *C3*, *PAX8*) and ciliated cell markers (*FOXJ1*, *PIFO*). Earlier studies have reported linear differentiation trajectories of FT epithelium (Dinh *et al.*, 2021; Ulrich *et al.*, 2022). Dinh *et al.* construct a linear trajectory using pseudotime analysis, where secretory clusters differentiate to ciliated cells via a population of ‘uncharacterised cells’, which closely resemble our populations of Pre-Ciliated-1 and Pre-Ciliated-2. Conversely, Ulrich *et al.* describe two epithelial progenitor populations where a ‘Peg Cell’ differentiates to secretory cells, and an ‘EMT progenitor’ gives rise to ciliated cells. Significantly increasing the number of cells analysed and utilizing RNA velocity analysis allowed us to map a bidirectional differentiation model where the Progenitor population forms were either Mature Secretory or Mature Multiciliated cells. This differentiation coincides with the menstrual phases, where the mature populations are only present in the secretory phase. Previous studies have demonstrated that the FT does not undergo cyclical proliferation, but it is hormonally responsive (Maclean *et al.*, 2020); these results suggest it may indeed undergo cyclical maturation and differentiation.

We validated our development model using RNA velocity confidence and magnitude, which previous FT differentiation trajectory studies have not done. Based on the principles of gene expression dynamics, chaotic gene expression suggests a progenitor or pluripotent stem cell type (Furusawa and Kaneko, 2009). In the ‘Progenitor’ population, high velocity magnitude indicates a faster differentiation trajectory, and low velocity confidence indicates velocity vectors are not correlated between neighbouring cells and are therefore more chaotic, suggesting a pluripotent cell type (Rue and Martinez Arias, 2015; Bergen *et al.*, 2020). Development of stability and loss of chaos in gene expression is also associated with loss of pluripotency and terminal differentiation (Zipori, 2004). This is seen in the mature epithelial populations, where velocity has a smaller magnitude and higher confidence compared to the progenitor population. This suggests that differentiation trajectories have slowed, and there is a cohesive agreement between neighbouring cells in the direction of velocity vectors, or development.

*In vitro* studies have supported this FT epithelial cell differentiation model where a secretory-like progenitor cell gives rise to ciliated and mature secretory cells. Yamamoto *et al.* (2016) characterized FT epithelial cells from primary human adult and foetal FT cells using clonal isolation techniques. These stem cells did not express *LGR5*, or secretory or ciliated markers *FOXJ1* or *PAX2* and underwent differentiation in a trans-well air–liquid interface culture to form secretory and ciliated cells (Yamamoto *et al.*, 2016), suggesting that a pluripotent population of *LGR5-*FT epithelial cells exists, similar to our ‘Progenitor’ cells here. Assessing the progenitor population *in vitro* is critical to confirming this model of FT epithelial differentiation.

We compared secretory phase FT and endometrial epithelial cells to determine how they contribute to tissue-specific function. Previous studies have compared expression of individual transcripts and proteins between the two tissues (Amso *et al.*, 1994; Amir *et al.*, 2009; Duncan *et al.*, 2010; Tempest *et al.*, 2018; Maclean *et al.*, 2020), but there is no comparison of transcriptomes at a bulk or single-cell resolution. As observed across human tissues, ciliated cells in the FT and endometrium have highly similar transcriptomes (Ivliev *et al.*, 2012).

In contrast, large differences were seen between FT and endometrium secretory epithelial cells. By constructing hypergraphs to identify higher-order gene interactions (Johnson, 2011; Battiston *et al.*, 2021), we identified clusters of genes likely to underpin the functional tissue divergence. The widely used FT-specific marker OVGP1 was among these genes, suggesting that this secreted glycoprotein has functional roles in FT that have previously been ascribed to interactions with the zona pellucida that regulate fertilization and embryo development (Algarra *et al.*, 2016; Zhao *et al.*, 2022). LCN2, another secreted protein specific to the FT hypergraph, is expressed throughout the female reproductive tract and is canonically involved in iron transport. However, recent studies have suggested roles for LCN2 in regulating the innate immune system in the endometrium (Krizanac *et al.*, 2024). Lipid and cholesterol transport-associated genes were also identified as functional genes: *APOA1*, *STARD4*, *STX11*, *SYNRG*, *TTC39B.* Lipids are known to constitute a component of FT fluid and are thought to influence oocyte maturation and embryo development (Monisova *et al.*, 2007; Winuthayanon and Li, 2018). Several of these genes are also associated with intra- and extracellular vesicle transport, which has been described as a component of FT fluid (Hirst *et al.*, 2005; Dabrazhynetskaya *et al.*, 2012; Alminana *et al.*, 2017; Zhang *et al.*, 2022). These functional genes describe a secretory, lipid-rich environment facilitated by the highly specialized secretory cells of the FT.

As the hypergraph models identified genes previously shown to have functional roles in the FT, we wanted to assess whether genes relevant to tubal ectopic pregnancy, a common FT pathology, were also identified. Remarkably, of 22 tubal ectopic pregnancy GWAS genes present in the hypergraph DEGs, 13 were found to be in the most connected gene clusters, including the highest-scoring disease-associated gene, MUC1. Ranking these GWAS genes based on their row sum, or connectivity, in the hypergraph models offered a metric for functional importance and yielded other genes likely to be causally involved in tubal ectopic pregnancy.

Notably, *MUC1* was the second-highest-ranked GWAS gene in the hypergraphs, providing confidence that this metric was correlated to clinical and functional importance. Furthermore, this analysis may shed light on eutopic implantation, as other genes suspected of mechanistically supporting embryo attachment and implantation were present in the ectopic pregnancy GWAS and hypergraph central clusters of the FT and luminal endometrial epithelium: *CLDN1* and *PLXNA4* (Margarit *et al.*, 2010; Xu *et al.*, 2022; Wang *et al.*, 2024). The strong association of tubal ectopic pregnancy and implantation in the secretory cells of FT and endometrial luminal epithelium also suggests that specialized secretory cells mediate embryo attachment in health and disease. *HLA-DBR1* and *HLA-DRB5* were highly connected in the FT. HLA alleles have also been associated with recurrent implantation failure and miscarriage (Kruse *et al.*, 2004; Thomsen *et al.*, 2021).

When we examined expression levels of the ectopic GWAS genes, *SLC7A2* and *MUC1* were both expressed at the highest level in the Mature Secretory-1 FT population. Interestingly, this mature epithelial population is only present in the secretory phase. These specialized secretory cells may protect against ectopic embryo implantation in the secretory phase and ensure the embryo is transitioned to the embryo by providing a unique secretory environment with high levels of MUC1.

In summary, this work has demonstrated the utility of sophisticated meta-analyses of existing data sets to reveal novel insights into the tissue structure and dynamics of the FT. As well as yielding novel insights into the differentiation process of mature epithelial cell types, our approach has identified candidate genes of clinical relevance in the susceptibility of individuals to ectopic implantation.

## Supplementary Material

deaf200_Supplementary_Figure_S1

deaf200_Supplementary_Figure_S2

deaf200_Supplementary_Figure_S3

deaf200_Supplementary_Figure_S4

deaf200_Supplementary_Figure_S5

deaf200_Supplementary_Figure_S6

deaf200_Supplementary_Figure_S7

deaf200_Supplementary_Table_S1

deaf200_Supplementary_Table_S2

deaf200_Supplementary_Table_S3

deaf200_Supplementary_Table_S4

deaf200_Supplementary_Table_S5

deaf200_Supplementary_Table_S6

## Data Availability

FT scRNA-seq datasets are available at GEO under GSE183837 (Hu *et al.*, 2020), GSE151214 (Dinh *et al.*, 2021), and GSE178101 (Ulrich *et al.*, 2022). Endometrial scRNA-seq dataset is available at ArrayExpress under E-MTAB-10287 (Garcia-Alonso *et al.*, 2021). Code is available at https://github.com/adam-stevens-lab/fallopiantube.
