# Supplementary material for: Identification of novel differentiation trajectories and gene network associations with ectopic pregnancy in fallopian tube epithelium
Source: Hum Reprod. 2025 Nov 3;40(12):2369–81. doi: 10.1093/humrep/deaf200 (PMC12675418; doi:10.1093/humrep/deaf200)
Supplement: deaf200_Supplementary_Figure_S1 [file deaf200_supplementary_figure_s1.pdf]

OXFORD

human reproduction

Human Reproduction, 2025, 1–1
https://doi.org/10.1093/humrep/deaf200
Supplementary Data

Hypergraph construction

1. Count matrix

	Gene 1	Gene 2	Gene 3	Gene 4	Gene 5	Gene (n)
Cell 1	9	9	7	11	11	...
Cell 2	7	12	14	14	13	...
Cell 3	7	7	8	9	6	...
Cell 4	7	11	10	13	6	...
Cell (n)	...	...	...	...	...	...

Take the cell x gene count matrix, calculate standard deviation of each gene, and remove those with SD=0

2. Correlation matrix

	Gene 1	Gene 2	Gene 3	Gene 4	Gene 5	Gene (n)
DEG 1	9	9	7	11	11	...
DEG 2	7	12	14	14	13	...
DEG 3	7	7	8	9	6	...
DEG 4	7	11	10	13	6	...
DEG (n)	...	...	...	...	...	...

Create a correlation matrix of the set list of DEGs against the rest of the transcriptome genes

3. Incidence matrix

	Gene 1	Gene 2	Gene 3	Gene 4
DEG 1	1	0	0	0
DEG 2	1	0	0	0
DEG 3	0	1	0	0
DEG 4	0	1	1	0
DEG 5	0	1	1	1
DEG 6	0	0	1	0
DEG 7	0	0	1	1
DEG 8	0	0	0	1

Take the absolute values of the correlation matrix, binarise this against the standard deviation

4. Reduced adjacency matrix

	DEG 1	DEG 2	DEG 3	DEG 4	DEG 5	DEG 6	DEG 7	DEG 8
DEG 1	1	1	0	0	0	0	0	0
DEG 2	1	1	0	0	0	0	0	0
DEG 3	0	0	1	1	1	0	0	0
DEG 4	0	0	1	2	2	1	1	0
DEG 5	0	0	1	2	3	1	2	1
DEG 6	0	0	0	1	1	1	1	0
DEG 7	0	0	0	1	2	1	2	1
DEG 8	0	0	0	1	1	0	1	1

Perform a matrix multiplication against the transpose of the incidence matrix

Supplementary Figure S1. Method of hypergraph construction.
