# Supplementary material for: Identification of novel differentiation trajectories and gene network associations with ectopic pregnancy in fallopian tube epithelium
Source: Hum Reprod. 2025 Nov 3;40(12):2369–81. doi: 10.1093/humrep/deaf200 (PMC12675418; doi:10.1093/humrep/deaf200)
Supplement: deaf200_Supplementary_Figure_S2 [file deaf200_supplementary_figure_s2.pdf]

OXFORD
human reproduction

Human Reproduction, 2025, 1–1
https://doi.org/10.1093/humrep/deaf200
Supplementary Data

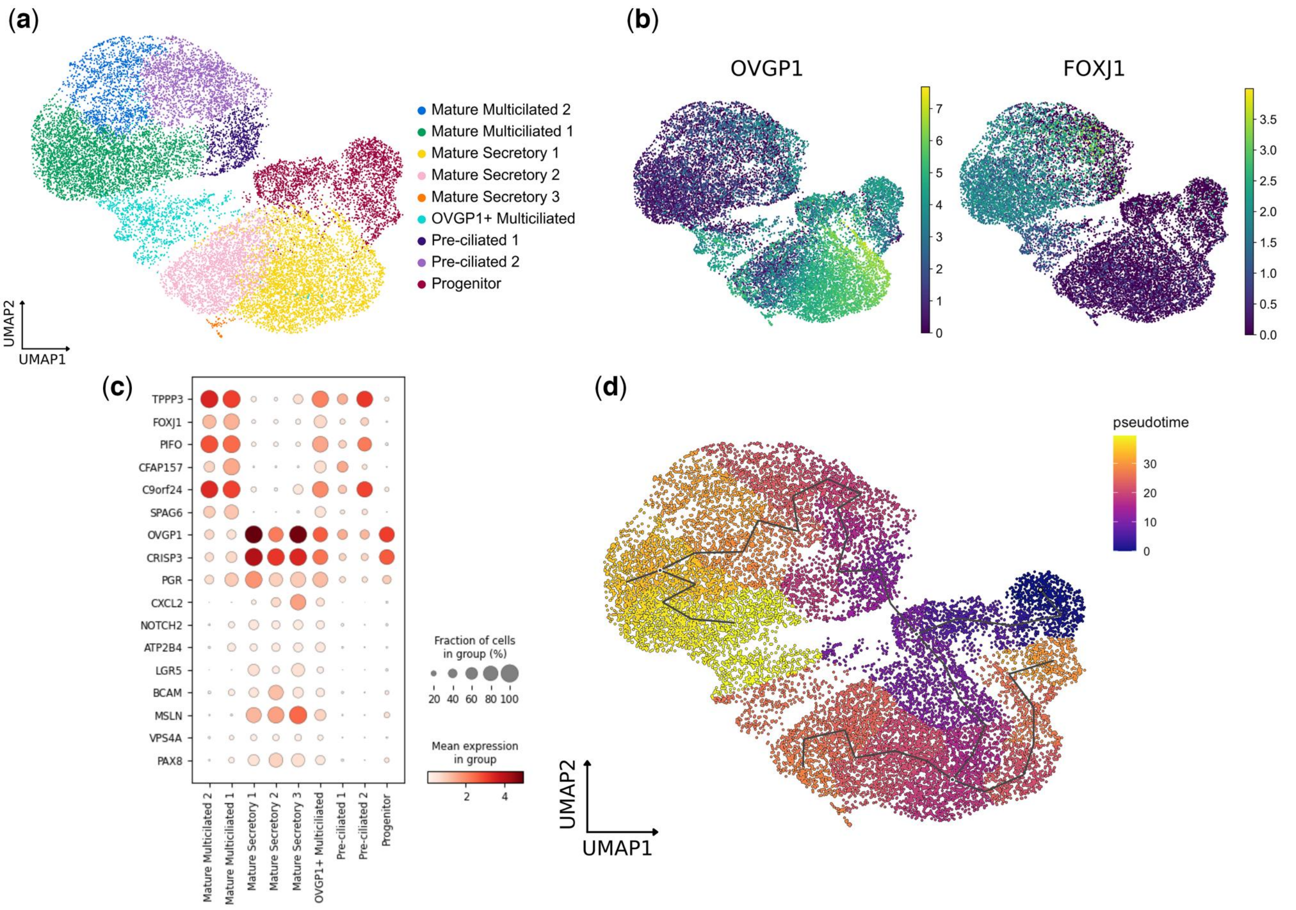

Supplementary Figure S2. Replication of cell clusters and differentiation trajectory in Weigert *et al.* (2025) validation dataset. (**a**) UMAP of 18 170 epithelial cells isolated from Weigert *et al.* (2025) fallopian tube scRNAseq dataset, coloured by clusters identified by the Fallopian tube scRNAseq meta-analysis. (**b**) UMAP of marker gene expression: FOXJ1—Ciliated, OVGP1—Secretory. (**c**) Dotplot illustrating mean gene expression and fraction of cells expressing key marker genes in populations of FT epithelial cells. (**d**) Monocle3 pseudotime of bidirectional differentiation trajectory from Progenitor population. UMAP, Uniform Manifold Approximation Projection; FT, fallopian tube.
