# Supplementary material for: Identification of novel differentiation trajectories and gene network associations with ectopic pregnancy in fallopian tube epithelium
Source: Hum Reprod. 2025 Nov 3;40(12):2369–81. doi: 10.1093/humrep/deaf200 (PMC12675418; doi:10.1093/humrep/deaf200)
Supplement: deaf200_Supplementary_Figure_S3 [file deaf200_supplementary_figure_s3.pdf]

Human Reproduction, 2025, 1–1
https://doi.org/10.1093/humrep/deaf200
Supplementary Data

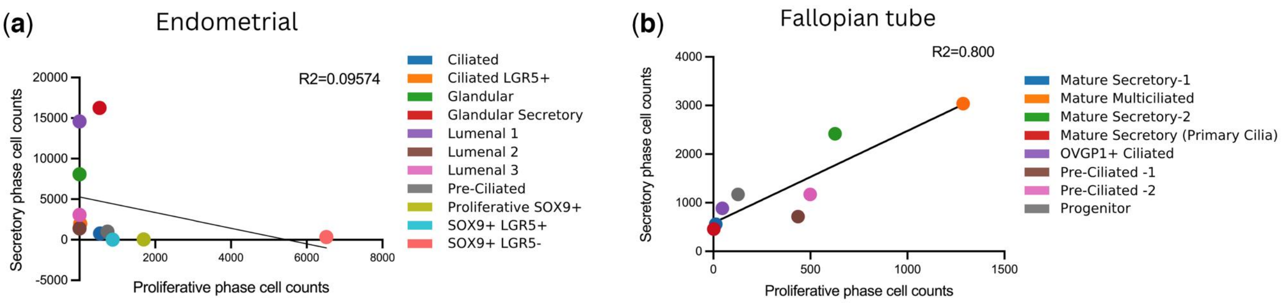

Supplementary Figure S3. Endometrial and fallopian tube (FT) epithelial cell population counts in the secretory versus proliferative menstrual phases. (**a**) Linear regression of endometrial cell counts between menstrual phases, $R2 = 0.09574$. (**b**) Linear regression of FT cell counts between menstrual phases, $R2 = 0.800$. FT, fallopian tube.
