# Supplementary material for: Identification of novel differentiation trajectories and gene network associations with ectopic pregnancy in fallopian tube epithelium
Source: Hum Reprod. 2025 Nov 3;40(12):2369–81. doi: 10.1093/humrep/deaf200 (PMC12675418; doi:10.1093/humrep/deaf200)
Supplement: deaf200_Supplementary_Figure_S4 [file deaf200_supplementary_figure_s4.pdf]

Human Reproduction, 2025, 1–1
https://doi.org/10.1093/humrep/deaf200
Supplementary Data

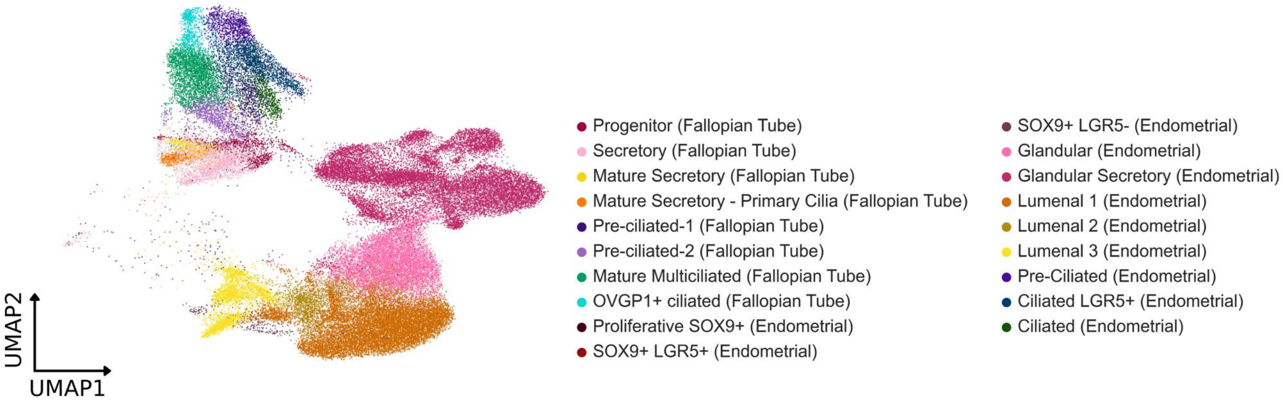

Supplementary Figure S4. UMAP of fallopian tube and endometrial epithelial cells in the secretory phase. All epithelial populations are shown. UMAP, Uniform Manifold Approximation Projection.
