# Supplementary material for: Identification of novel differentiation trajectories and gene network associations with ectopic pregnancy in fallopian tube epithelium
Source: Hum Reprod. 2025 Nov 3;40(12):2369–81. doi: 10.1093/humrep/deaf200 (PMC12675418; doi:10.1093/humrep/deaf200)
Supplement: deaf200_Supplementary_Figure_S5 [file deaf200_supplementary_figure_s5.pdf]

Human Reproduction, 2025, 1–1
https://doi.org/10.1093/humrep/deaf200
Supplementary Data

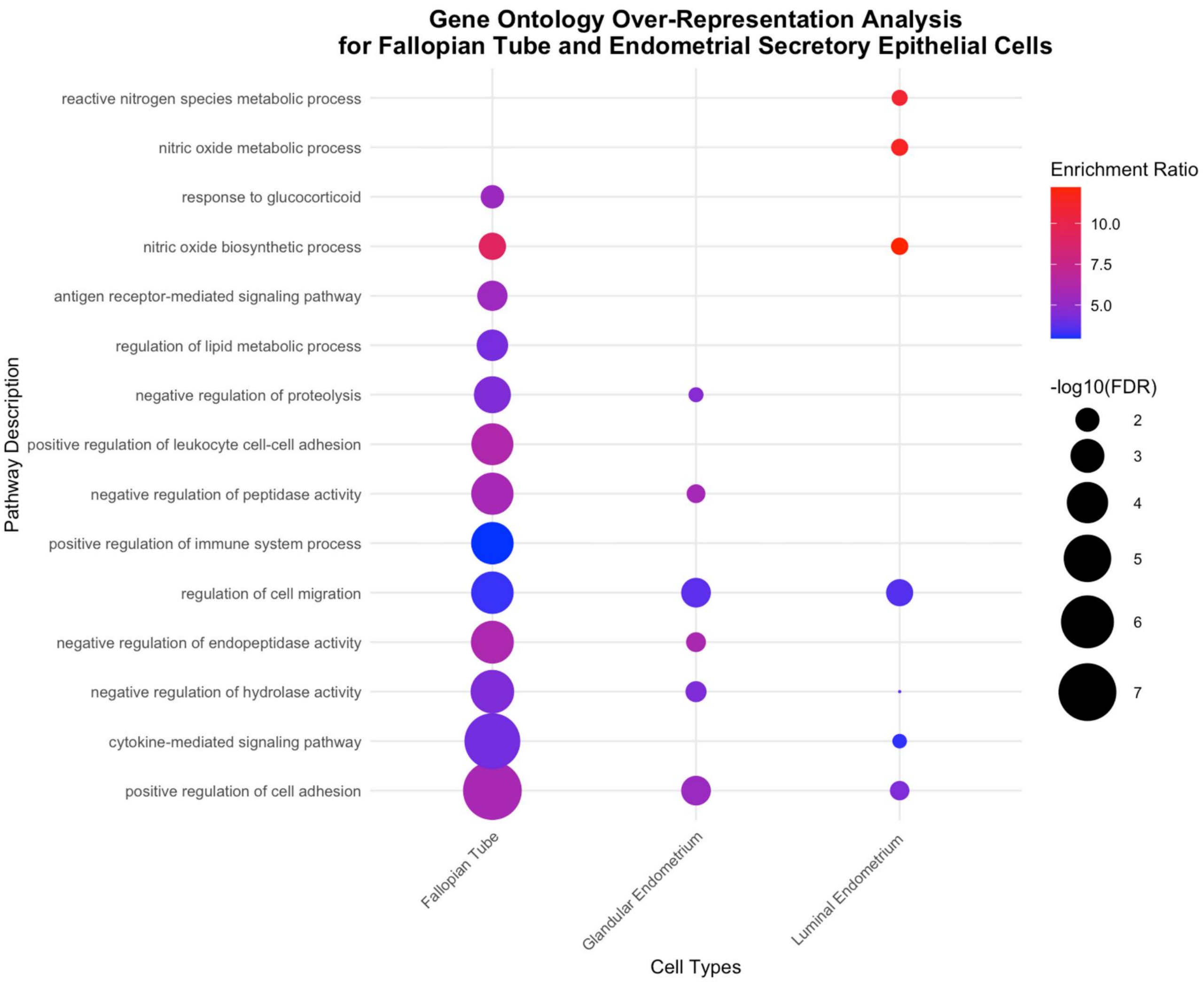

Supplementary Figure S5. Dot plot of gene ontology over-representation analysis using central clusters of fallopian tube and glandular and luminal endometrium hypergraphs. False discovery rate (FDR) is shown as −log10 and represents size of dots. Enrichment Ratio represents the colour of dots. FDR, false discovery rate.
