# Supplementary material for: Identification of novel differentiation trajectories and gene network associations with ectopic pregnancy in fallopian tube epithelium
Source: Hum Reprod. 2025 Nov 3;40(12):2369–81. doi: 10.1093/humrep/deaf200 (PMC12675418; doi:10.1093/humrep/deaf200)
Supplement: deaf200_Supplementary_Figure_S6 [file deaf200_supplementary_figure_s6.pdf]

Human Reproduction, 2025, 1–1
https://doi.org/10.1093/humrep/deaf200
Supplementary Data

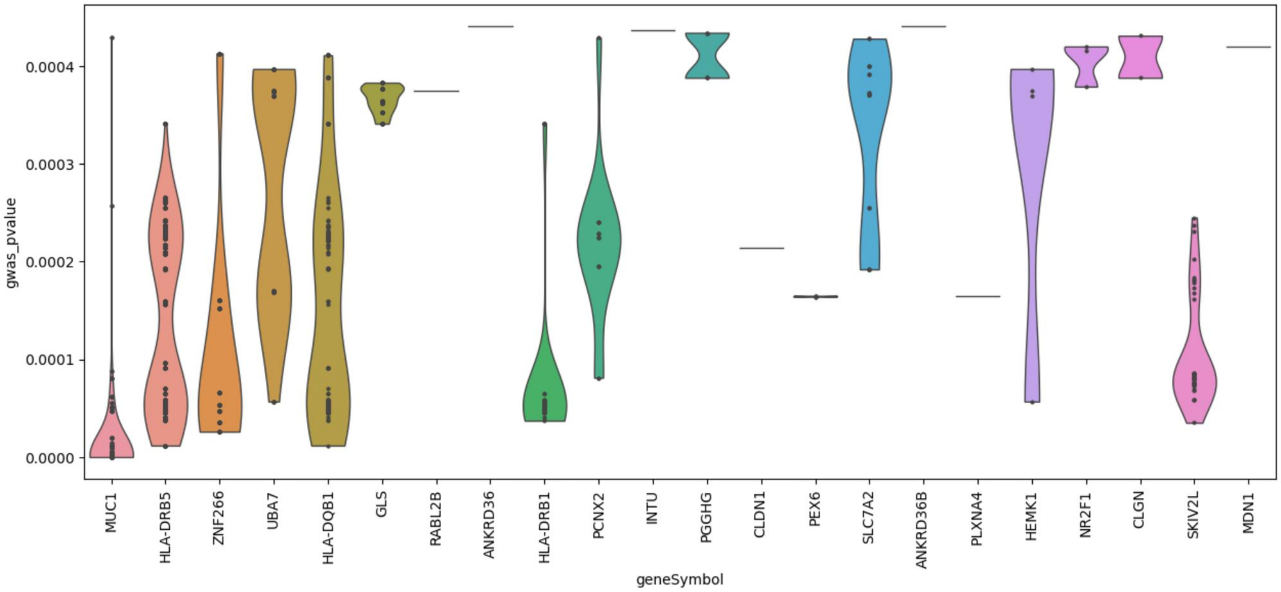

Supplementary Figure S6. Violin plot of the ectopic pregnancy GWAS genes P values. GWAS *P* values (*P* = 1.68 × 10–21–5.8 × 10–4) of genes in hypergraph models, obtained from summary statistics in GWAS catalogue (study accession GCST90272883); each point represents *P* value of individual rsID. GWAS, genome-wide association study; rsID, reference SNP cluster IDs.
