# Supplementary material for: Identification of novel differentiation trajectories and gene network associations with ectopic pregnancy in fallopian tube epithelium
Source: Hum Reprod. 2025 Nov 3;40(12):2369–81. doi: 10.1093/humrep/deaf200 (PMC12675418; doi:10.1093/humrep/deaf200)
Supplement: deaf200_Supplementary_Table_S1 [file deaf200_supplementary_table_s1.pdf]

OXFORD
human reproduction
Human Reproduction, 2025, 1–1
https://doi.org/10.1093/humrep/deaf200
Supplementary Data

Supplementary Table S1. Published patient and sample information.

Original study	Patient ID	Age	Cycle day	Menstrual stage	Section/sample type	Diagnoses
Dinh	Patient 1	47	3	Proliferative	Mid portion	Symptomatic fibroids, adenomyosis, endometriosis
Dinh	Patient 3	47	17	Secretory	Ampulla	Symptomatic fibroids
Dinh	Patient 4	41	7	Secretory	Mid portion	Symptomatic fibroids, adenomyosis, endometriosis
Dinh	Patient 5	33	31	Secretory	Mid portion	Symptomatic fibroids
Dinh	Patient 6	31	13	Proliferative	Ampulla, infundibulum, fimbriae	Uterine didelphys
Dinh	Patient 7	46	23	Secretory	Mid portion, fimbriae	Symptomatic fibroids
Dinh	Patient 8	62	–	Post menopause	Fimbriae	Symptomatic fibroids, endometrial polyps
Ulrich	Patient 9	46	28	Secretory	Fimbriae, ampulla, isthmus	Fibroid
Ulrich	Patient 10	52	37	Secretory	Fimbriae, ampulla, isthmus	Fibroids, AUB, dysmenorrhoea
Hu	Patient 11	50	–	Post menopause	Infundibulum	Adenomyosis, leiomyoma, inactive endometrium
Hu	Patient 12	45	–	Post menopause	Infundibulum	Adenomyosis, secretory endometrium, benign Brenner tumour
Hu	Patient 13	64	–	Post menopause	Infundibulum	Left ovarian benign fibroma
Hu	Patient 15	55	–	Post menopause	Infundibulum	Persistent vaginal spotting and discharge; benign vaginal inclusion cyst
Garcia-Alonso	Patient 16	37	–	Proliferative	Full thickness uterine wall	–
Garcia-Alonso	Patient 17	23	–	Secretory	Full thickness uterine wall	–
Garcia-Alonso	Patient 18	29	25	Secretory	Endometrial biopsy	–
Garcia-Alonso	Patient 19	26	20	Secretory	Endometrial biopsy	–
Garcia-Alonso	Patient 20	26	8	Proliferative	Endometrial biopsy	–
Wang	Patient 21	–	16	Proliferative	Endometrial biopsy	–
Wang	Patient 22	–	26	Secretory	Endometrial biopsy	–
Wang	Patient 23	–	20	Secretory	Endometrial biopsy	–
Wang	Patient 24	–	26	Secretory	Endometrial biopsy	–
Wang	Patient 25	–	29	Secretory	Endometrial biopsy	–
Wang	Patient 26	–	23	Secretory	Endometrial biopsy	–
Wang	Patient 27	–	20	Secretory	Endometrial biopsy	–
Wang	Patient 28	–	22	Proliferative	Endometrial biopsy	–
Weigert	D1_2509	47	–	Luteal	Fimbriae, ampulla, isthmus	–
Weigert	D2_3508	43	–	Follicular	Fimbriae, ampulla, isthmus	–
Weigert	D3_3586	37	–	n/a	Fimbriae, ampulla, isthmus	–
Weigert	D4_3604	43	–	n/a	Fimbriae, ampulla, isthmus	–
Weigert	D5_3619	37	–	Follicular	Fimbriae, ampulla, isthmus	–
Weigert	D7_3643	42	–	Follicular	Fimbriae, ampulla, isthmus	–
Weigert	D8_3668	48	–	n/a	Fimbriae, ampulla, isthmus	–
Weigert	D9_3669	44	–	Luteal	Fimbriae, ampulla, isthmus	–
Weigert	D10_3679	40	–	Luteal	Fimbriae, ampulla, isthmus	–
